# Gastrointestinal catastrophe after ingestion of liquid nitrogen-treated candy: a case of Dragon’s breath-induced perforation and postoperative complications

**DOI:** 10.1093/jscr/rjag005

**Published:** 2026-01-25

**Authors:** Hafiz Syed Zaigham Ali Shah, Ayesha Mahmood, Hassan Bin Aziz, Syed Salman Hussain Zaidi, Muhammad Musaab

**Affiliations:** King Edward Medical University/Mayo Hospital, Lahore, Punjab 54590, Pakistan; Aberdeen Royal Infirmary/University of Aberdeen, Foresterhill Road, Aberdeen AB25 2ZN, Scotland; King Edward Medical University/Mayo Hospital, Lahore, Punjab 54590, Pakistan; Aberdeen Royal Infirmary/University of Aberdeen, Foresterhill Road, Aberdeen AB25 2ZN, Scotland; King Edward Medical University/Mayo Hospital, Lahore, Punjab 54590, Pakistan; King Edward Medical University/Mayo Hospital, Lahore, Punjab 54590, Pakistan; King Edward Medical University/Mayo Hospital, Lahore, Punjab 54590, Pakistan

**Keywords:** liquid nitrogen, dragon’s breath candy, gastric perforation, barotrauma, erosive gastritis

## Abstract

Liquid nitrogen-infused ‘Dragon’s Breath’ snacks have gained popularity despite their potential to cause serious gastrointestinal injury. We report the case of a 14-year-old boy who presented to the emergency department of a tertiary care hospital, two hours after ingestion of ‘*Dragon’s Breath*’ *candy* with severe abdominal pain and distension leading to generalized peritonitis and sepsis. Abdominal X-ray showed massive pneumoperitoneum, raising suspicion of hollow viscus perforation. Emergency laparotomy performed, and there was a 3 × 2 cm gastric perforation along the lesser curvature. Primary repair was performed with drain placement, and the initial postoperative course was uneventful, leading to discharge on day 6. Three days later, he re-presented with hematemesis, melena, and haemoglobin of 8 g/dl. Upper gastrointestinal endoscopy showed mild erosive gastritis without active bleeding. This case highlights the significant morbidity associated with liquid nitrogen ingestion and emphasizes the need for safety regulations and postoperative endoscopic follow-up.

## Introduction

Liquid nitrogen has been used as a coolant for processing of beverages and for storage of food items [1]. Recently, its novel use has emerged to produce frozen foods and vapour clouds, commonly known as ‘dragon’s breath’ snacks [1]. Cases have been reported with accidental ingestion of liquid nitrogen along with such snacks, causing Gastrointestinal perforation [2]. We report a case of gastric perforation that developed post-operative complication after discharge from the hospital.

## Case report

We present a case of a 14-year-old boy, referred to the emergency department of a tertiary care hospital from a peripheral center, 2 hours after consuming liquid nitrogen–infused *‘Dragon’s Breath’ candy.* The patient reported sudden onset of severe abdominal pain, predominantly in the epigastric region, accompanied by progressive distension. Informed consent for publication of this case was obtained from the patient’s father due to his minor status.

**Figure 1:**
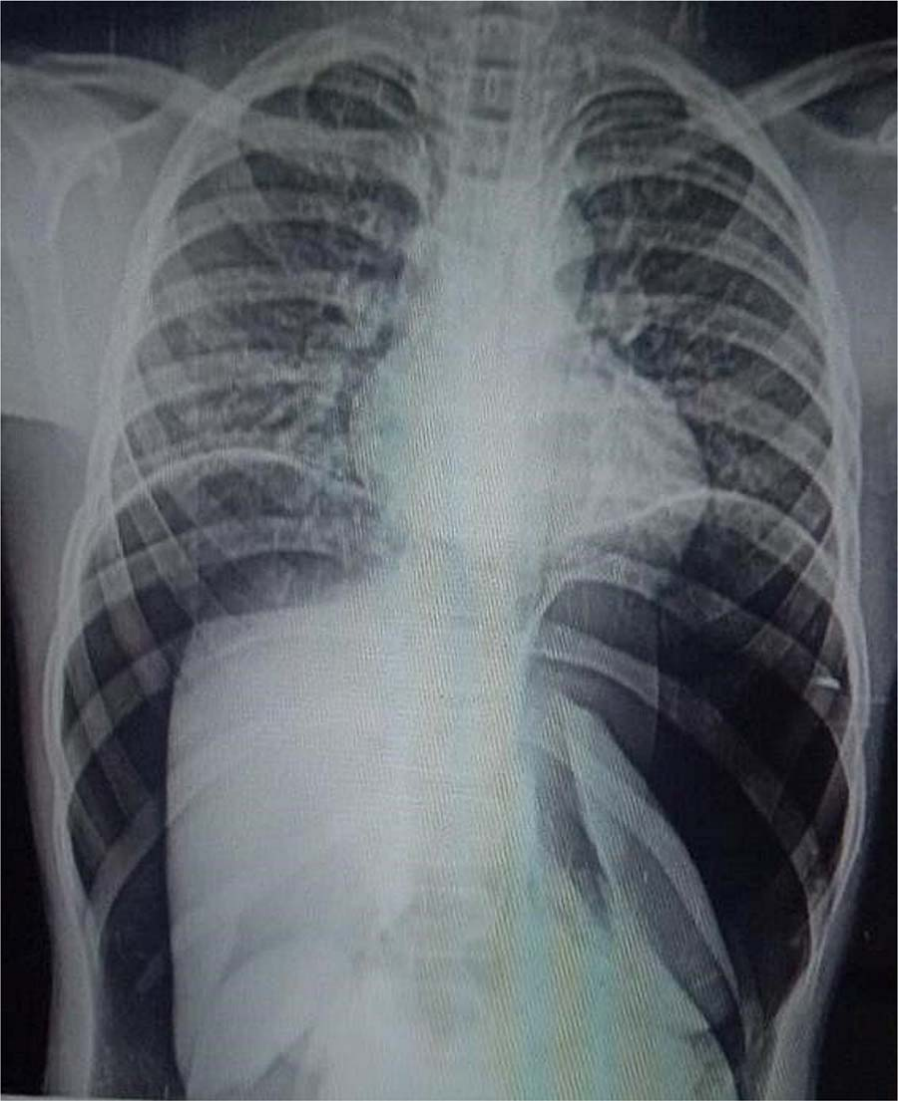
Chest X-rays with pneumoperitoneum.

**Figure 2:**
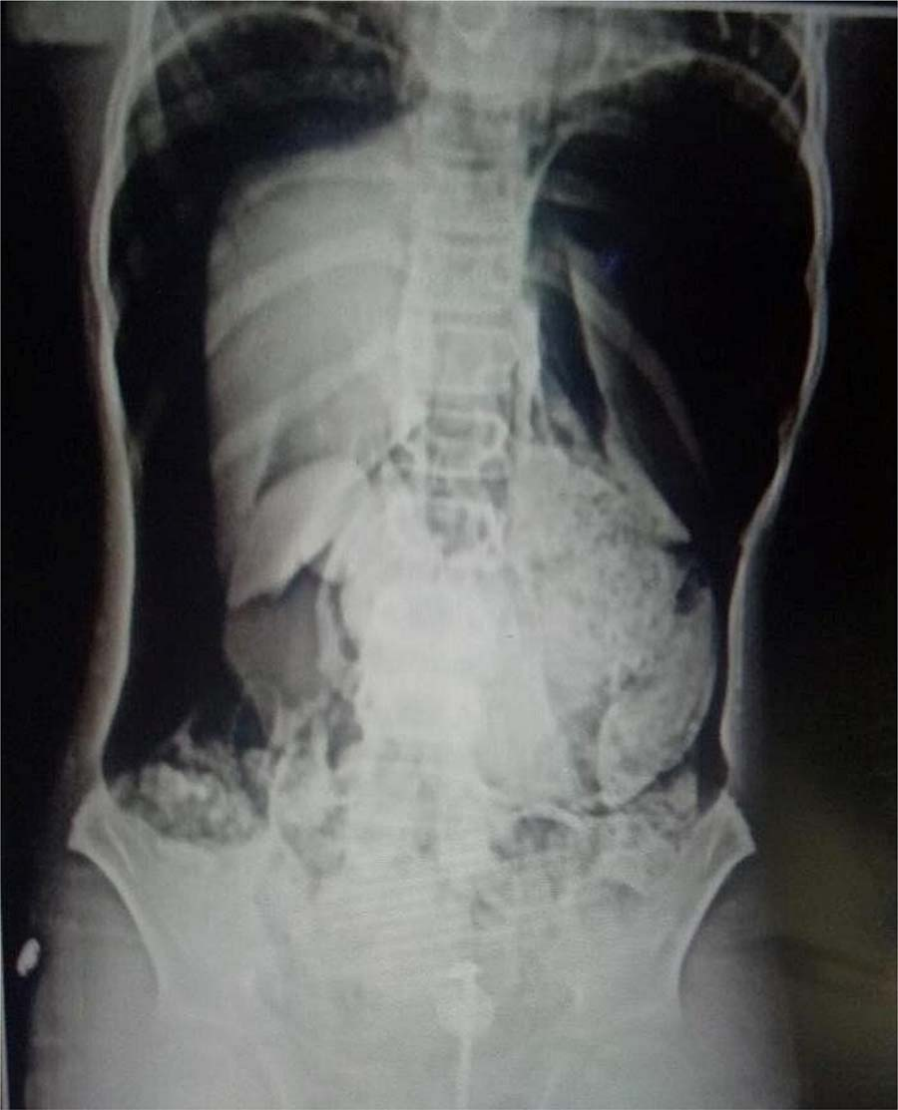
Abdominal X-rays with massive pneumoperitoneum.

On arrival, he was alert but visibly distressed due to pain. His vital signs were stable initially, but on examination, he had signs of generalized peritonitis with tense, diffusely tender abdomen in all quadrants and absent bowel sounds. Digital rectal examination was unremarkable. Initial laboratory investigations showed a total leukocyte count of 9.3 × 10^3^/μl and platelet count of 143 × 10^3^/μl. A plain abdominal X-ray demonstrated massive pneumoperitoneum (Figs 1 and 2), strongly suggestive of hollow viscus perforation. Based on clinical and radiological findings, a provisional diagnosis of *hollow viscus perforation* was made.

**Figure 3:**
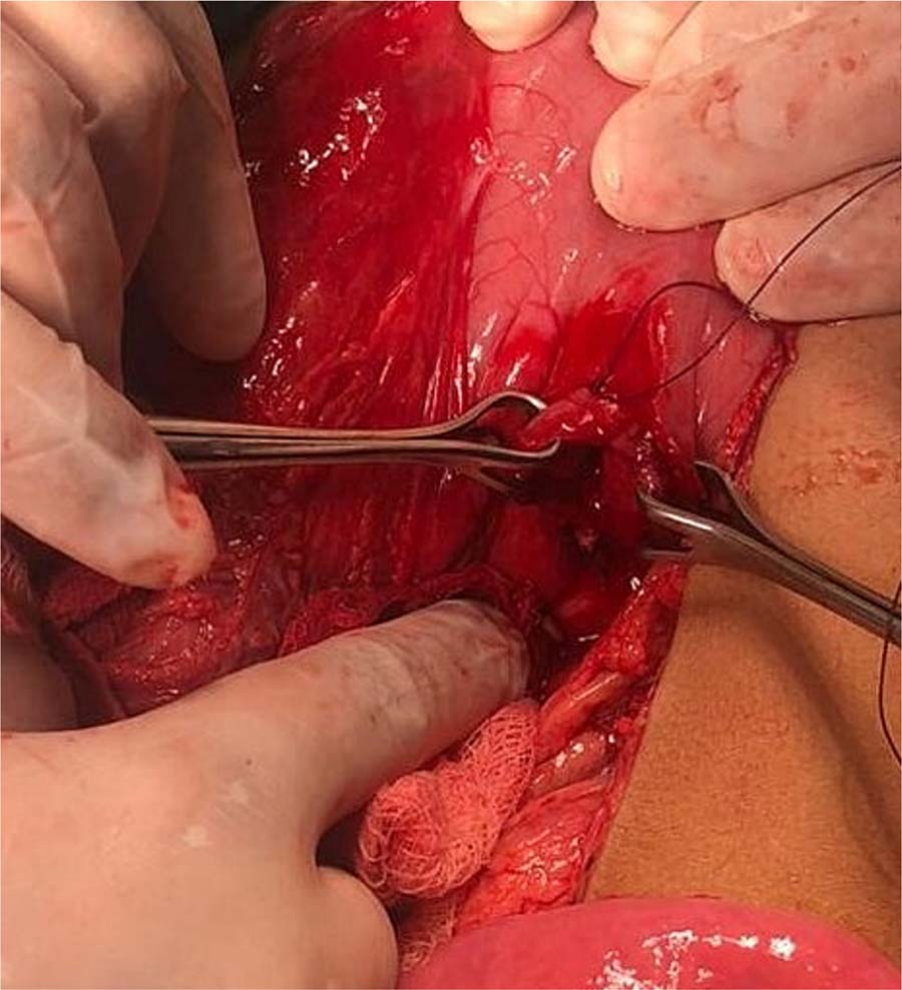
Gastric perforation at lesser curvature.

He underwent emergency laparotomy. A large gush of air upon entering the peritoneal cavity, confirming free intraperitoneal gas, and there was a perforation measuring approximately 3 × 2 cm was identified along the lesser curvature of the stomach, near the *incisura angularis* (Fig. 3). Primary repair was performed in a double-layer pattern. A drain was placed in the lesser sac to monitor postoperative leakage or bleeding. Postoperatively, he transferred to the High Dependency Unit (HDU) for monitoring. His remaining recovery was uneventful, and he was discharged on day 6.

Three days later, he presented to the emergency room with shock and complaints of hematemesis and melena with haemoglobin of 8 g/dl. He was promptly resuscitated and received blood transfusions to stabilize his haemoglobin. An urgent upper gastrointestinal endoscopy was performed to identify the source of bleeding. Endoscopic evaluation revealed mild *erosive gastritis* but no active bleeding site, ulceration, or evidence of recurrent perforation. It was managed conservatively and later on he was discharged with the follow up plans after 14 days.

## Discussion

Liquid nitrogen has been used in food and beverage industries since 1800s [1]. It has a very low boiling point(−196ºC) and evaporates rapidly at room temperature with a ratio of 1:694, that is, 1 ml liquid nitrogen converts to 694 ml of nitrogen gas. This explains barotrauma due to ingestion of liquid nitrogen. Absence of injury to oesophagus, as seen per op and later on confirmed with upper gastrointestinal endoscopy, showed barotrauma as possible mechanism of injury and massive pneumoperitoneum [2].

Considering previous studies, the site of perforation can be explained by Laplace’s law, which states that larger the sphere radius, greater the tension required to withstand internal pressure. In stomach, body is the most distensible part, with its lesser curvature most susceptible to perforation, as in our case, due to its relative immobility because of neighbouring celiac trunk and gastroesophageal junction. In our case, perforation was found along lesser curvature of stomach, which was similar to previous cases reported [3, 4].

One aspect that differs in this case is post-operative complication in the form of upper GI bleed. Previous studies didn’t show any gastritis or bleed on follow-up upper GI endoscopy [4]. Patient presented back with the hematemesis, and endoscopy showed erosive gastritis. Previous studies didn’t show any post-operative complications except for sepsis in one case [5].

Liquid nitrogen is being increasingly used in snacks and drinks for creating smog effect. However, precautions must be observed for its use [6]. Liquid nitrogen also causes thermal burns when comes in contact with body surface. Cases have been reported, causing thermal burns leading to amputations due to contact with liquid nitrogen [7].

## Conclusion

There should be high-level safety measures by industries at national and international levels in developing countries for use of liquid nitrogen, particularly for children and teenagers who are not aware of these hazards. This case highlights the serious morbidity in the form of gastrointestinal perforation associated with liquid nitrogen–treated foods and beverages. Post-operative complications cannot be ignored in these cases, and postoperative upper GI endoscopy should be done in every case.
